# Notes on Medical Matters and Medical Men in London

**Published:** 1846-08

**Authors:** David W. Yandell

**Affiliations:** London


ART. IV.—*Notes on Medical Matters and Medical Men in London.* By DAVID W. YANDELL, M.D.

In my last letter I said a few words about England's great surgeon, Robert Liston, but I had only time then to give my first impressions concerning this eminent man. In returning to him and dwelling more minutely on his character and history, I hope you will not consider that I am spending too much time upon one individual. If you could see the honor and respect with which Mr. Liston is treated by his colleagues, pupils, and the medical profession at large, and then see him among his patients and at the operating table, and more than all, witness his kindness to our young countrymen, you would not believe that I could easily say too much about him. Professor Gibson gives a characteristic description of Liston in "his Rambles in Europe"—of his passion for rowing, and for domestic animals, particularly "his enormous black cat *Tom*, who was not unfrequently mounted alongside his master in the splendid chariot, and a constant guest at his hospitable board"—of his tall, robust, and elegantly formed person; his handsome features and penetrating eye; his modesty, playfulness, and benevolence. Dr. G. had been told that he was rough and uncouth in his manners, "a perfect *ursa major*"—"a mere operator or carver without judgment or discretion;" but his quick eye soon saw in him a surgeon who, "ere long, would rise to the top of the profession" in the first city in the world. Mr. Liston is certainly eccentric; but the prejudices which were excited by his eccentricities have yielded to the sterling qualities which shine in him as a surgeon and a gentleman. He retains his fondness for rowing, which in his youth was so great that his father put him to the study of anatomy in order that he might be fitted for a nautical life, if his passion for the sea should continue after he had completed his professional studies. He did not go to sea, however, and his time is now so entirely employed by professional duties, that little leisure is left him for indulging in his favorite recreation.

Robert Liston was born at Linlithgowshire, in Scotland, in 1795, and consequently is now in the fifty-second year of his age. He commenced his studies preparatory to the life which he proposed following with Mr. Barclay, at that time professor of anatomy in the University of Edinburgh. His progress in anatomy was so rapid that he was soon appointed demonstrator for the pupils of Barclay, and in this capacity he continued to labor for six years, during the latter two of which he was house surgeon to the Edinburgh Hospital. After this he visited London, and under the direction of Mr. T. Blizzard entered the London hospital. In 1816, being just twenty-one years of age, he took his degree in the College of Surgeons. The year following he returned to Edinburgh, where he taught anatomy and, for several years, surgery to a private class of more than a hundred and fifty students. He also received the appointment of assistant surgeon to the Edinburgh College, and after discharging the duties pertaining to that office with striking ability for nine months he was made chief surgeon, in which capacity he remained during his residence in Edinburgh. In 1834 he came to London where he has resided, engaged in a growing business, ever since. In 1835 he was elected surgeon to the University College Hospital, and made professor of Clinical Surgery in the University. Besides various papers in medical journals, and a Memoir on Hernia, he has written two works on Surgery which are well known in our country, one of them having gone through several editions, with valuable notes by your distinguished colleague, Professor Gross, and the other having been issued under the supervision of Professor Mütter, who has also made valuable additions to the original. They stand in no need of any praise which I could bestow upon them, having found their way into the libraries of all who take an interest in the practice or progress of surgery. Mr. Liston is still sound and vigorous in mind and body, with the promise of many years before him—with even a youthful ardor in the accumulation of knowledge, we may yet expect to see many valuable contributions from his pen to our stock of surgical literature. At this season of the year he is not engaged in lecturing, so that I have not had many opportunities of forming an opinion of his powers in that way, but judging from the remarks I have a few times heard him make after his operations, I should say that he is not a fluent speaker, and that his aim is rather to give a clear statement of facts bearing upon his subject, than to appear graceful or eloquent. I have seen him execute several operations, the most important of which was amputation of the thigh by the circular method—a mode quite unusual with him, his uniform practice having been to perform the flap operation. It was one of those cases in which no display of peculiar skill could be made, but even in this Mr. Liston showed his greatness; and he did so not so much by the use of his knife, as by the general order and plan of the whole procedure. There was no noise, no bustle; every one, surgeons and assistants, had been taught his duties and his place. While the patient was being placed on the table for the operation, Liston was in another room changing his coat for an old one, which is his custom instead of putting on an apron. Presently he made his appearance, glanced at the bandages and the position of the patient, adjusted the tournequet, and then took up the knife and began the operation in the quietest manner imaginable. After he had finished the amputation he applied most of the ligatures himself, adjusted the edges of the wound, gave a few directions about the treatment, made some remarks concerning the cause and nature of the operation, and, with a simple nod of the head, walked out of the room.

I am in London at the worst season of the year for the prosecution of my studies by means of lectures. The winter term is over, and the great men, with a few exceptions, have withdrawn from their chairs until October, when their labors begin again, to terminate in April. Graham, Quain, Sharpey, Taylor, and Williams, esteemed among the most able teachers in London, are not now lecturing. A teacher with whom I am very much pleased is Mr. Murphy, author of a work on Parturition of great merit, which I hope will soon be republished in America. He is a plain, unassuming man, about forty-five years of age, of quiet manners and appearance. As a lecturer he would not strike you. His voice is feeble, his speech slow and indistinct, and his manner embarrassed and uninteresting; but his language is correct and scholarlike, simple and concise, and his matter excellent. As a man you are obliged to like to him; his face is expressive of great amiability and kindness, and is a true index to his character. I breakfasted with him, and found him pleasant in conversation, and exceedingly obliging. He is regarded as one of the first accoucheurs in Great Britain.

Professor Grant, one of the most distinguished of the scientific men of England, is delivering a course of lectures on zoology. He is fifty or fifty-five years old, about five feet ten inches high; his head is large and well formed; his eyes are blue, more nearly grey; his nose is sharp, small, and well turned; his mouth is that of a speaker, large and wide; his manner of lecturing, though somewhat diffuse, is very good, being graceful and agreeable. He warms up as he gets into his subject, and, in a lecture I heard him deliver, spoke with as much fire about the habits of the oyster, and the circulation of a lobster, as our eminent friend, Prof. D., would do before the "Physiological Temperance Society."

The students attending the hospitals and lectures in London have none of the affability so characteristic of young men in our country; neither are they so fine in appearance as those you are accustomed to see. They are earnest, assiduous students, but distant and indifferent; crowding around their teachers, eager to hear, careless whether standing in your way or not, and looking all the time most ludicrously frigid. Students of the same small class will often be found wholly unknown to each other. There are those I have seen who have followed Mr. Liston and other teachers through the hospitals for three or four years, and expect to take their degrees soon, without ever having exchanged salutations with them. I know a class consisting of five students dissecting for Mr. Liston, who meet every morning, and have dined together, on an average, twice a week for two months, who, nevertheless, profess no acquaintance. I inquired of one of them, a day or two ago, the name of a gentleman, pointing to one of his class-mates. "Well, I declare I don't know," was his reply. But they are students, in truth. You may walk into the library room of the University College and find twenty or thirty young men poring over their books, from which they are taking notes, not one of whom will raise his head to see who you are. You ask the librarian for the book you wish to consult; it is handed to you immediately, and you take your seat at one of the tables without your next neighbor's turning his eyes to see whether you are an acquaintance or a stranger. There you may occupy yourself all the morning, and never hear a word spoken above a whisper. Every student attending the University College takes copious notes. The lecture rooms are well adapted to this purpose, each seat having on its back a plank of suitable width and height to form a desk for writing. The note-books generally used are well bound, of convenient size to be carried in the pocket, and look as if they were intended for preservation and future reference. When the hospitals are visited by the attending physician or surgeon, you see the best students with their note-books in their hands ready to write down whatever may be said respecting the cases, and copy out the prescriptions which are placed upon a board at the head of each bed.

The relations between teacher and pupil here are by no means such as exist in the United States. The surgeons talk to their dressers in the hospitals in a way that not many of our students could bear—bear, I mean, without injured feelings. They treat their pupils as boys of ten and fifteen years are treated with us by the roughest pedagogues. There is no social intercourse—no conversation in the door-way and on the steps of the College—no walking into the room of a professor to while away the hour occupied by one of his dull colleagues in turning over his books and preparations, or "probing" him about your chances for a diploma. No; there is nothing of all this between the English medical student and his teacher. A simple elevation of the hat on the part of the pupil, and an ambiguous touch of the beaver by the professor, makes the sum of their social intercourse, unless indeed the student chances to be a "dresser" in a hospital. In that case, if a bandage should be awkwardly applied, or an ulcer found in a filthy condition, or there be a patient in a bed of whose case the dresser happens to know nothing, ten to one, "stupidity;" "carelessness;" "without excuse;" "any one should have known better;" "who ever heard of such a thing!" "what were you doing all day, that you could not attend to this?" will greet his ears in much less time than it has taken me to write down the brief, emphatic sentences, which sound strangely enough to one unused to this style of address.

I send you two cases which I met with in a late number of the Dublin Quarterly Journal, (see page 153) in the first of which I feel more than ordinary interest because there is at this time in the University College Hospital a case analogous to it—aneurism of the radial artery, caused by a gun-shot wound of the hand, which Mr. Liston commenced treating, by pressure, a short time before my arrival, of which I will make a report when the patient is discharged. The following are the particulars of a case of *femoral hernia* which I saw treated in the same hospital.

M. W., a washerwoman, æt. 43 years, was admitted into the hospital on the morning of the 18th of May, with femoral hernia of the right side. Six years before she had been operated upon by Mr. Liston for hernia occurring in the same spot; from that time she had constantly worn a truss which, although it occasionally allowed a portion of the bowel to protrude, prevented its coming down to a dangerous extent. Two days previously to her admission, while lifting some heavy weight, she felt the bowel pass down in larger bulk than usual, and was not able as on other occasions of the kind to return it. When I first saw her, the tumor occupied nearly the whole of the inner surface of the the thigh, and was as large as a fœtal head; it was slightly painful and tender to the touch, and had produced some constitutional disturbance.

The patient was placed in a warm bath with a view to relaxing the muscular system; while she was in the bath attempts were made at reduction by the taxis; but these failed until the patient by her own efforts returned the bowel, which ascended with a gurgling noise. The finger could be passed up through the crural rings; no stricture of the bowel could be detected, showing that the reduction had been complete. An anodyne and a cathartic enema were ordered.

19th. The patient passed a restless night; countenance anxious; tongue coated; pulse quick and feeble; breathing hurried and performed entirely by the muscles of the chest; pain and tenderness upon pressure in the lower part of the abdomen and along the whole course of Poupart's ligament. She was ordered xv leeches to the abdomen; pills of calomel grs. ij, pulv. opii gr.$\frac{1}{4}$, every four hours; cathartic enema.

20th. Patient slept none; pulse 120, and feeble; tongue coated in the middle and red at the tip and edges; thirst excessive; pain and exquisite tenderness over the abdomen; countenance extremely anxious. Repeat the pills every two hours; ten leeches, afterwards a linseed meal poultice to the abdomen; cathartic enema.

21st. The patient is evidently sinking; pulse weak, and 140; the same group of symptoms but greatly exaggerated; during the night patient vomited a dark colored matter, and had several bloody stools. Ordered blister to the abdomen; wine, and pills of calomel grs.iv and opium gr.$\frac{1}{4}$ every hour; mercurial ointment and olive oil ää ℥ij; of this ʒij to be rubbed into the abdomen every alternate hour. The tumor has returned several times but was easily reduced, with the same gurgling noise; the finger could be readily passed into the abdomen through the enlarged rings, and at no time could any strictured portion of the bowel be detected. At 6 P.M. the patient died, vomiting a matter of the color and appearance of coffee grounds.

*Post-mortem.*—This revealed nothing save the evidence of peritonitis and enteritis throughout almost the whole of the abdomen, and the congested condition of the bowel which had been protruded. There was no appearance of stricture in any part of the intestines. The greater part of the small intestine presented, here and there, patches of lymph, and in all parts an amount of congestion which as effectually blocked up the alimentary canal as a stricture could have done. I watched the progress of this case, saw it treated by Dr. Quain, and witnessed the post-mortem examination with great interest. The propriety of an operation in order to determine whether or not there was stricture, I heard suggested. The inflammation which led to the fatal result was doubtless produced by the too powerful efforts, made by the patient herself, to reduce the hernia previous to her admission into the hospital.

London, May 25th, 1846.

